# PIK3R1 fusion drives chemoresistance in ovarian cancer by activating ERK1/2 and inducing rod and ring-like structures

**DOI:** 10.1016/j.neo.2024.100987

**Published:** 2024-03-14

**Authors:** Heidi Rausio, Alejandra Cervera, Vanina D. Heuser, Gun West, Jaana Oikkonen, Elena Pianfetti, Marta Lovino, Elisa Ficarra, Pekka Taimen, Johanna Hynninen, Rainer Lehtonen, Sampsa Hautaniemi, Olli Carpén, Kaisa Huhtinen

**Affiliations:** a Institute of Biomedicine and FICAN West Cancer Centre, Faculty of Medicine, University of Turku, Turku, Finland; b Drug Research Doctoral Programme (DRDP), University of Turku, Turku, Finland; c Research Program in Systems Oncology, Research Programs Unit, Faculty of Medicine, University of Helsinki, Helsinki, Finland; d Genómica Computacional, Instituto Nacional de Medicina Genómica, Mexico City, Mexico; e Department of Engineering, Enzo Ferrari, University of Modena and Reggio Emilia, Modena, Italy; f Department of Pathology, Turku University Hospital, Turku, Finland; g Department of Obstetrics and Gynecology, Turku University Hospital and University of Turku, Turku, Finland; h Department of Pathology, University of Helsinki and HUSLAB, University Hospital, Helsinki, Finland

**Keywords:** High-grade serous ovarian cancer, Fusion gene, Rods and rings (RRs), Platinum resistance

## Abstract

Gene fusions are common in high-grade serous ovarian cancer (HGSC). Such genetic lesions may promote tumorigenesis, but the pathogenic mechanisms are currently poorly understood. Here, we investigated the role of a PIK3R1-CCDC178 fusion identified from a patient with advanced HGSC. We show that the fusion induces HGSC cell migration by regulating ERK1/2 and increases resistance to platinum treatment. Platinum resistance was associated with rod and ring-like cellular structure formation. These structures contained, in addition to the fusion protein, CIN85, a key regulator of PI3K-AKT-mTOR signaling. Our data suggest that the fusion-driven structure formation induces a previously unrecognized cell survival and resistance mechanism, which depends on ERK1/2-activation.

## Introduction

High-grade serous cancer (HGSC) is the most common and lethal ovarian cancer subtype with limited treatment options [1]. The driving genomic alterations in HGSC are *TP53* mutations and homologous recombination deficiency, which lead to dysfunctional DNA repair mechanisms and genetic instability [2]. The tumors are characterized by extensive copy number alterations and structural chromosomal aberrations, such as gene fusions [3], [4], [5]. The genomic instability and tumor heterogeneity make HGSC challenging to treat when the tumors have developed resistance to conventional platinum-taxane chemotherapy and PARP inhibitors [6].

A gene fusion occurs when two separate genes merge due to translocation, deletion, insertion, or inversion [7]. Genes can also fuse at the RNA level when two pre-mRNAs merge, resulting in a fusion transcript [8,9]. Although both gene and transcript fusions are common in HGSC, recurrent gene fusions are rare [4,[10], [11], [12]]. The unique fusions may underlie common oncogenic mechanisms. However, there are only a few studies on the fusions’ functional role. Some recent studies suggest that fusions may function as biological determinants of drug resistance in HGSC, such as SLC25A40-ABCB1 [13]. In a previous study, we identified fusions in 107 HGSC tumors and found that fusions are enriched in the PI3K-AKT-mTOR pathway genes [5]. Furthermore, multiple studies have shown that the PI3K-AKT-mTOR pathway is essential and commonly hyperactivated in HGSC [2,14,15]. In this study, we focused on the functional role of PIK3R1-CCDC178 fusion initially identified in the ovarian cancer tissue and lymph node metastasis of an HGSC patient.

*PIK3R1* in chromosome 5 encodes the p85α protein, which is a regulatory subunit of PI3 kinase (PI3K) and has an important role in multiple cellular processes, including proliferation, migration, and cisplatin resistance [16,17]. p85α contains SH3, BH and three SH2 protein domains. In regulating the PI3K-AKT-mTOR pathway, SH2 binding domains are required to stabilize the catalytic subunit of PI3K, p110α, encoded by the *PIK3CA* gene. SH2 domains are also needed for ligand-induced phosphorylation of receptor tyrosine kinases, after which the inhibitory effect of the p85α on the p110α is released. This, in turn, leads to activation of PI3K-AKT-mTOR signaling. The PTEN phosphatase can antagonize the pathway to which p85α binds with its SH3 and BH domains [16,18,19]. *PIK3R1* is rarely mutated in HGSC [2], and the pathway-activating mutations of the PI3K-AKT-mTOR pathway typically involve *PTEN* and *PIK3CA* [2,11,20,21]. However, *PIK3R1* is frequently mutated in many other cancers [22]. *PIK3R1* mutations have diverse impacts on cellular signaling, e.g. ERK/MAPK cascade, cellular phenotypes, and treatment responses [23], [24], [25], [26], [27].

In addition to PTEN, the PI3K-AKT-mTOR signaling is regulated by two adaptor proteins Cbl-interacting 85-kDa protein (CIN85) and its paralog CD2-associated protein (CD2AP) [28,29]. CIN85 and CD2AP contain three SH3 domains and proline-rich regions [30]. CIN85 can negatively regulate the PI3K-AKT-mTOR signaling cascade by binding on the SH3 domain of p85α [31,32]. In contrast, CIN85 activates RAS-MEK1/2-ERK1/2 signaling [33] and contributes to cell adhesion, migration, and invasive behavior [34], [35], [36]. High expression of CIN85 has been discovered in lymph node metastasis in breast cancer and esophageal squamous cell carcinoma; however, the precise mechanisms by which CIN85 contributes to the malignant phenotype remain unclear [37,38].

Because p85α inhibits PI3K activity, we hypothesized that the dysfunctional PIK3R1-CCDC178 fusion protein could promote activation of the PI3K-AKT-mTOR signaling cascade and further promote tumor growth, invasiveness, and drug resistance. Our results show that overexpression of the fusion induces HGSC cell migration and increases platinum resistance but, unexpectedly, the mechanism is unrelated to PI3K-AKT-mTOR pathway. Instead, the fusion protein induces elevated expression of CIN85 and activation of the ERK pathway. Platinum resistance is associated with formation of structures containing both the fusion protein and CIN85. Morphologically the structures resemble rods and rings (RRs), which have earlier been described as a resistance mechanism to ribavirin treatment [39].

## Materials and Methods

### Cell culture

The high-grade ovarian serous adenocarcinoma cells, OVCAR-8 (National Cancer Institute Frederick Cancer DCTD Tumor and Cell line repository), and the human embryonic kidney cells, HEK293 (American Type Culture Collection, Manassas, VA), were cultured in RPMI-1640 media (Gibco, Thermo Fisher Scientific) supplemented with 10 % fetal bovine serum (FBS, Biowest), 2 % ultraglutamine (Lonza) and 1 % penicillin-streptomycin (Gibco, Thermo Fisher Scientific). All cells were maintained at 37°C in a humidified 5 % CO2 atmosphere.

### Plasmids

PIK3R1-CCDC178_pcDNA3.1(+)-CeGFP (GeneScript Biotech) overexpression plasmid contains optimized PIK3R1-CCDC178 fusion gene sequence and enhanced green fluorescent protein (eGFP). The insert was removed from the PIK3R1-CCDC178_pcDNA3.1(+)-CeGFP plasmid using NheI, BamHI, and XbaI restriction enzymes and ligated according to sticky-end ligation protocol (Thermo Fischer Scientific) to create control plasmid. PIK3R1-CCDC178_pcDNA3.1(+)-CeGFP and pcDNA3.1(+)-CeGFP plasmids were transformed into competent *E. coli* using heat shock. Bacteria were grown on agar plates (Fisher BioReagents) containing 100 µg/ml ampicillin (Fisher BioReagents) and incubated overnight at 37°C. Isolated colonies were grown in LB broth (Fisher BioReagents) overnight at 37°C and selected with 100 µg/ml ampicillin. DNA was extracted using a Nucleospin Plasmid QuickPure kit according to the manufacturer´s protocol. Plasmid purity was verified using gel electrophoresis and Sanger sequencing.

### Transfections

PIK3R1-CCDC178_pcDNA3.1(+)-CeGFP and pcDNA3.1(+)-CeGFP plasmid DNA were transfected into OVCAR-8 cells using Lipofectamine 2000 (Invitrogen). HEK293 cells were transfected using FuGene reagent (Promega) according to the manufacturer´s instructions and selected using 150-300 µg/ml Geneticin (Invitrogen). Cells were sorted by FACS to create stably expressing cell lines. Cell lines were cultured in RPMI-1640 media supplemented with 10 % fetal bovine serum, 2 % ultraglutamine, 1 % penicillin-streptomycin, and 50 µg/ml Geneticin. All in vitro experiments were performed with stably expressing GFP-control vector or PIK3R1-CCDC178-GFP fusion cell lines in Geneticin-free media. Cells were regularly tested for mycoplasma using a MycoALert PLUS Mycoplasma detection kit (Lonza).

### Colony assay

2000 cells were seeded on 12-well plates. After five days, cells were washed with PBS and incubated with different treatments for 72 h (Supplementary Methods Table S1). Next, cells were washed with PBS, stained with crystal violet dye for 10 mins, and washed with tap water three times. Finally, the stain was extracted using 1 % SDS. The absorbance was measured at 600 nm wavelength using Victor2 1420 Multilabel Counter. Three independent experiments were performed.

### Cell viability

10 000 cells were seeded and incubated overnight. Cells were treated with 5 µmol/L cisplatin, 3 µmol/L trametinib, and their combination for five days. Absorption was measured at 490 nm every 24 hours using CellTiter 96 AQueous One Solution*.*

### Cell migration assay

30 000 cells per well were seeded to obtain confluent density on 96-well IncuCyte ImageLock plates (Essen Bioscience) and incubated overnight. Cell monolayers were scratched using the IncuCyte 96-well WoundMaker Tool (Essen Bioscience), washed once with PBS, and replaced with fresh media. Cells were imaged every second hour for 72 hours in the IncuCyte ZOOM imaging device. Three independent experiments were performed.

### Immunofluorescence staining

Coverslips were coated with Geltrex. Cells were seeded with wanted density and incubated overnight. RRs were induced with mycophenolic acid (MPA) for 4 hours and with 6-Diazo-5-oxo-L-norleucine (DON) for 24 hours. Cells were fixed with 4 % paraformaldehyde (PFA) for ten min and washed with PBS. Cells were permeabilized and blocked with 0,5 % Triton and 5 % bovine serum albumin (BSA) in PBS for 30 min at room temperature. Primary antibodies were incubated overnight at 4°C. Coverslips were washed with PBS and secondary antibody incubation for 1,5 hours at room temperature (List of used antibodies in the Supplementary Methods Table S2). ProLong Diamond Antifade Mountant with DAPI (Thermo Fisher Scientific) was used for mounting. Fluorescence images were taken using Invitrogen EVOS M5000 Imaging System (Thermo Fisher Scientific) and confocal images by 3i Spinning Disc. Representative middle Z-stack sections are shown in the confocal images.

### Correlative light-electron microscopy (CLEM)

Cells were plated on 35 mm Petri dishes with gridded glass bottoms (MatTek). Cells were treated with 5 µmol/L cisplatin for 96 hours. The medium was removed, and cells were fixed for 10 min with 4 % PFA in 0.2 M HEPES buffer (pH 7.4), prewarmed to +37°C. PFA was removed, and 0.2 M HEPES was added. After the initiative fixation, phase contrast and fluorescence images were taken using Invitrogen EVOS M5000 Imaging System (Thermo Fisher Scientific). Next, cells were fixed with 2 % glutaraldehyde in 0.2 M HEPES (pH 7.4) for two hours at room temperature and stored in 0.2 M HEPES at +4°C overnight. Cells were then washed with 0.2 M HEPES twice and postfixed with 1 % osmium tetroxide containing 1.5 % potassium ferrocyanide for one hour. Next, cells were washed with 0.2 M HEPES twice for five min. Dehydration was performed with 70 %, 95 %, and 100 % ethanol at +4°C for one minute in each concentration and finally with 100 % ethanol for 30 min at room temperature. The cells were then incubated in a mixture of Epon resin and 100 % ethanol for 30 min and finally in 100 % Epon for two hours. Beem capsules filled with Epon were set upside down on top of the samples, guided by the grid markings in the glass bottom of the dish. After 36 hours of incubation at +60°C, capsules were removed from the dishes, and the blocks were trimmed to expose the cells of interest for thin sectioning. Sections were cut using a diamond knife and collected on Pioloform-coated one-slot grids. Imaging was performed using a JEM-1400Plus transmission electron microscope.

### Immunoprecipitation

Control and PIK3R1 fusion cells were cultured in T75 flasks. At 90 % confluence, cells were detached with Trypsin and centrifuged at 1000 rpm for 5 min. Cell pellets were lysed with Lysis buffer (Chromotek), which included proteinase and phosphatase inhibitors (Thermo Scientific). Lysates were incubated on ice for 30 minutes with periodic mixing. Cell debris was removed by centrifugation at 17,000 × g for 10 minutes at 4°C. 50 µl of the supernatant was saved as an input fraction. The GFP-tagged fusion protein complex was immunoprecipitated by GFP-Trap Dynabeads (Chromotek). 5x volumes of bead slurry (125 µl) and cell pellets (5x T75) were used per sample. Equilibrated beads were rotated with the diluted lysate for 1,5 h at 4°C. Beads were separated with a magnet and washed twice with Wash buffer (Chromotek). Proteins were eluted with 2x Laemmli according to the manufacturer´s protocol. Elution was achieved by adding 50 µl of Acidic Elution Buffer (Chromotek) to the sample, which was then subjected to constant up-and-down pipetting for 60 seconds at room temperature. The resulting eluate was neutralized by adding 5 µl of Neutralization Buffer (Chromotek). Samples were run on SDS-gels 200V, 45 min. Gels were stained overnight with PageBlue Protein Staining Solution (Thermo Fisher Scientific) and washed with Milli-Q water for 4 h (Supplementary Figure S1 A). Proteins were left on the beads for the LC-ESI-MS/MS Analysis.

### LC-ESI-MS/MS Analysis

Mass spectrometry analyses were performed at the Turku Proteomics Facility supported by Biocenter Finland. The LC-ESI-MS/MS analyses were performed on a nanoflow HPLC system (Easy-nLC1000, Thermo Fisher Scientific) coupled to the Q Exactive HF mass spectrometer (Thermo Fisher Scientific, Bremen, Germany) equipped with a nano-electrospray ionization source. Peptides were first loaded on a trapping column and subsequently separated inline on a 15 cm C18 column (75 μm x 15 cm, ReproSil-Pur 3 μm 120 Å C18-AQ, Dr. Maisch HPLC GmbH, Ammerbuch-Entringen, Germany). The mobile phase consisted of water with 0.1 % formic acid (solvent A) or acetonitrile/water (80:20 (v/v)) with 0.1 % formic acid (solvent B). A linear 20 min gradient from 6 to 39 % of eluent B, followed by a wash stage with 100 % of eluent B, was used to eluate peptides. The MS data were automatically acquired using Thermo Xcalibur 4.1 software (Thermo Fisher Scientific). An information-dependent acquisition method consisted of an Orbitrap MS survey scan of mass range 350–1750 m/z followed by HCD fragmentation for the 10 most intense peptide ions. Protein data can be found in Supplementary Table S3.

### Western blot

Control-GFP and PIK3R1-CCDC178-GFP expressing OVCAR-8 and HEK293 cells were lysed with M-PER mammalian protein extraction reagent (Thermo Scientific) supplemented with lysis buffer protease and phosphatase inhibitor (Pierce, Thermo Scientific). Samples were incubated for 2 hours at 4°C, centrifuged at 17 000 rcf for 30 min at 4°C, and the supernatant was collected. Western blotting was performed using 4-20 % sodium dodecyl-sulfate polyacrylamide gel electrophoresis (SDS-PAGE) gels, and samples were run at 200V for 45 min. Proteins were transferred to 0.2 µm nitrocellulose membranes (Bio-Rad Laboratories, Inc.) by Trans-Blot Turbo Transfer System (Bio-Rad Laboratories, Inc.) and blocked with 5 % milk for 1 h. Membranes were incubated with the primary antibody overnight at 4°C, washed three times for five minutes with TBST, incubated for 1 h with the secondary antibody, and rewashed three more times in TBST. Blots were detected using an ECL blotting substrate (Pierce ECL Western Blotting Substrate, Thermo Scientific, and SuperSignal West Femto Maximum Sensitivity Substrate, Thermo Scientific).

### Sequencing data

Detected fusion was studied at the DNA level through whole-genome sequencing. Four fresh frozen tumor samples and a blood cell control were sequenced with HiSeq X Ten (Illumina, USA) as 150bp paired-end sequencing. The median coverage for these 5 samples was 28. Data were aligned to GRCh38.d1.vd1. Because fusion was not detected earlier with specific tools, breakpoints were inspected through Baseplayer [40] from aligned data nearby the expected fusion breakpoints. Breakpoints were considered with at least three reads over all four tumor samples and no reads at blood cell control [15].

## Results

In this study, we focused on the functional role of PIK3R1-CCDC178 fusion initially identified by genome-wide RNA sequencing in two out of three tumors of an HGSC patient: a lesion in the right ovary and in metastasis of a right para-aortic lymph node but not in another right ovarian tumor lesion [5]. In this study, we further confirmed the presence of the fusion in these organs by an independent mechanism, RNA *in situ* hybridization (Supplementary Fig. S2 A & B and Supplementary Methods). The present study investigated the functional role of the PIK3R1-CCDC178 fusion in the stably overexpressing OVCAR-8 HGSC cell line. The main results were confirmed in HEK293 cells (Supplementary Fig. S3).

### *PIK3R1 fusion induces cell motility and resistance to cisplatin and trametinib*

**Fig. 1:**
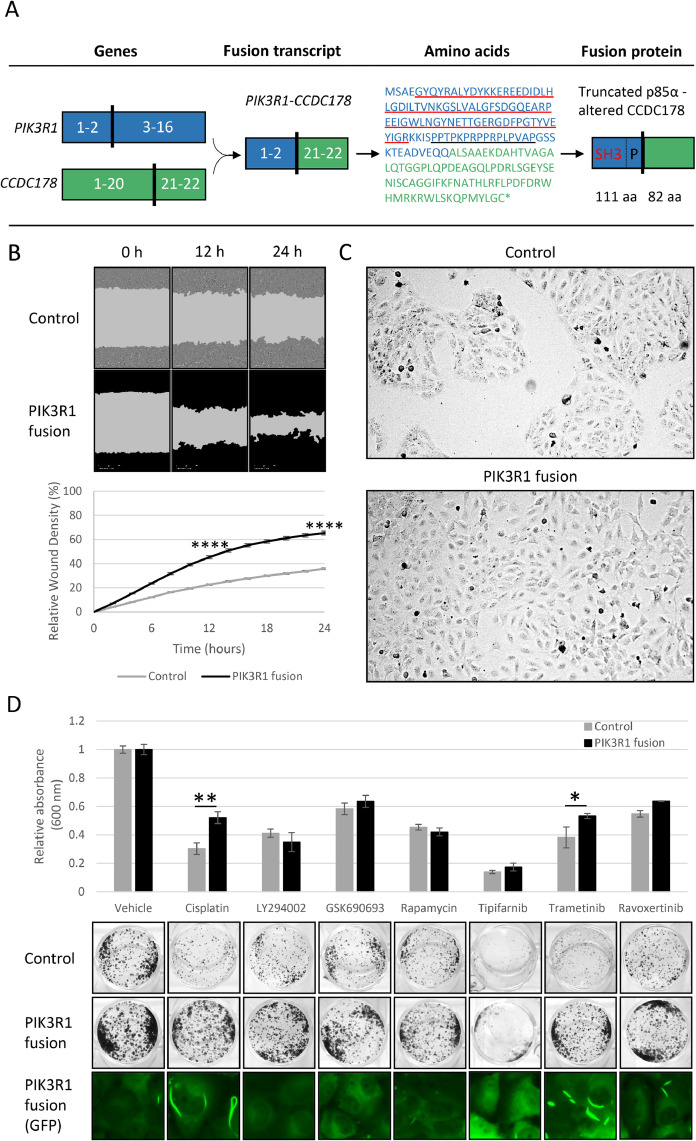
**PIK3R1 fusion induces cell motility and drug resistance.** A) Schematic representation of PIK3R1-CCDC178 fusion and its fusion protein. The fusion contains the first two exons of PIK3R1 and the last two exons of the CCDC178 with a reading frame shift. Numbers depict the exons. PIK3R1 fusion protein consists of a truncated p85α, including the SH3 protein domain (red) and a proline-rich region (black), with an altered c-terminal CCDC178 sequence. Protein domains are marked with corresponding colors underlined in the amino acid sequence. B) Migration of vector control and PIK3R1 fusion cells detected by wound healing assay. C) Cell morphologies under 10x magnification by phase contrast microscopy. D) Cells response to platinum and pathway inhibitors in colony formation assay. Cells were grown 8 d without treatment or treated with cisplatin, LY294002 (PI3Ki), GSK690693 (pan-AKTi), rapamycin (mTORi), tipifarnib (Rasi), trametinib (MEKi) and ravoxertinib (ERKi) for 3 d starting on day 5. Data are normalized to the vehicle. Fluorescence images at the bottom row indicate the expression of protein complexes in PIK3R1 cells under different treatments. Statistical analysis by unpaired t-test; *p ≤ 0.05, **p ≤ 0.01, ***p ≤ 0.001, ****p ≤ 0.0001. Error bars represent ±SEM.

*PIK3R1-CCDC178* fusion consists of the first two exons of *PIK3R1* in chromosome 5 and the last two exons of *CCDC178* in chromosome 18*. CCDC178* gene encodes a coiled-coil protein 178, whose physiological and pathological role is largely unknown [41,42]. The translated fusion protein contains a truncated p85α, including an SH3 domain and a proline-rich region. The c-terminal tail of the fusion protein is formed of an 82 amino acids long sequence, which does not represent CCDC178 protein due to reading frame shift (Fig. 1A). The sequence analysis did not recognize conserved protein domains in the fusion's c-terminus [43].

Based on the p85α function, we hypothesized that PIK3R1 fusion activates PI3K-AKT-mTOR signaling cascade, which can occur via the lack of the inhibitory effect on the p110α subunit or via defective interaction with PTEN phosphatase due to a lack of SH2 domains. We first assessed the effect of the PIK3R1 fusion protein on OVCAR-8 cell proliferation and migration. We examined proliferation by analyzing phase object confluence with Incucyte, but found no noticeable difference between control and PIK3R1 fusion cells. Furthermore, a Western blot analysis was performed to assess proliferating cell nuclear antigen (PCNA) levels, further confirming the absence of differences in proliferative activity between the cell lines (data not shown). PIK3R1 fusion cells presented significantly enhanced motility in wound healing assay, indicating that fusion can promote invasion and metastatic dissemination (Fig. 1B & Supplementary Fig. S4). Morphologically, PIK3R1 fusion cells formed unorganized populations while vector expressing control cells grow in round and compact colonies (Fig. 1C). These morphological features were also evident in the PIK3R1 fusion-overexpressing HEK293 cells (Supplementary Fig. S3 A).

The fusion's influence on the PI3K-AKT-mTOR pathway was first defined by the pathway inhibitor's effect on cell viability. Also, the inhibitors of the interacting RAS-MEK1/2-ERK1/2 pathway and platinum, the first line chemotherapy drug in HGSC, were included. Interestingly, PIK3R1 fusion cells presented with elevated resistance to cisplatin (Fig. 1D). PIK3R1 fusion cells also showed increased resistance to a MEK1/2 inhibitor trametinib but not to PI3K or AKT inhibitors (Fig. 1D & Supplementary Fig. S5). Upon fluorescence microscopic analysis, we noticed that GFP-tagged PIK3R1 fusion formed distinctive complexes specifically in cells treated with cisplatin or trametinib. Furthermore, treatment resistance correlated with the number and size of the structures (Fig. 1D & Supplementary Fig. S6).

### *ERK1/2 is activated in the PIK3R1 fusion cells*

**Fig. 2:**
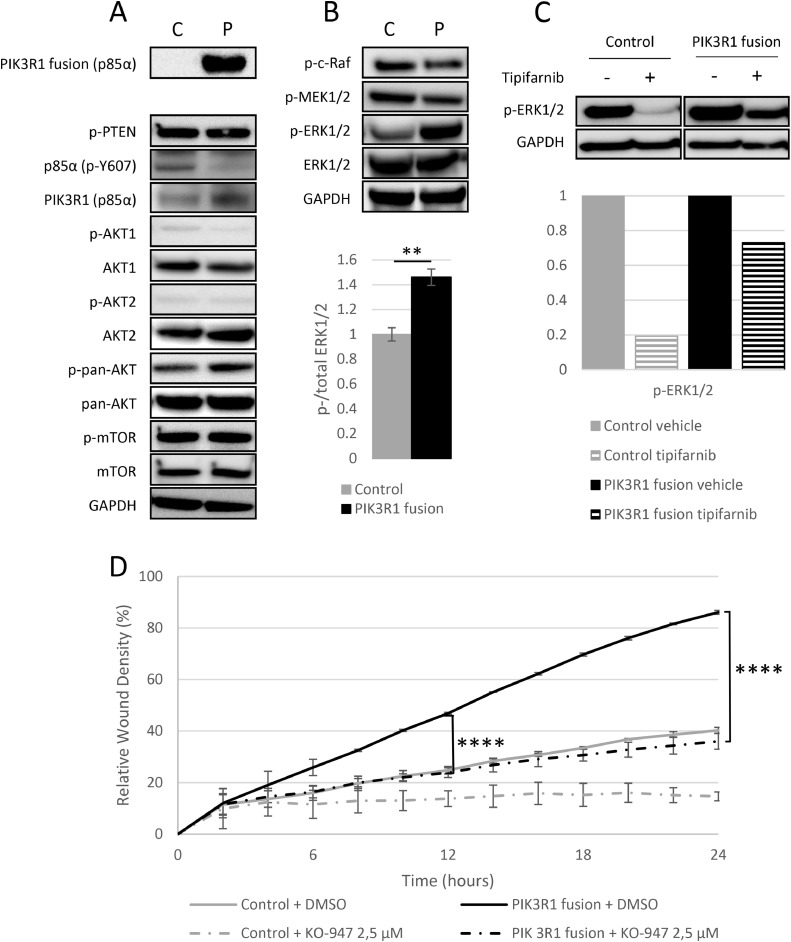
**ERK1/2 is activated in the PIK3R1 fusion cells.** A) Protein expression of the PI3K-AKT-mTOR pathway shows decreased phosphorylation of native p85α in the fusion expressing cells (P) than in vector control cells (C), while the other pathway proteins remained unaltered. B) Instead, ERK1/2 phosphorylation is elevated in the fusion expressing cells, and C) remains phosphorylated even with 10 µmol/L Ras inhibitor tipifarnib for 12 h. D) Migration of both vector control and PIK3R1 fusion cells without and with KO-947 (ERKi) by wound healing assay. Data are represented as mean ± SEM of three independent experiments for Western blot analyses and two independent experiments ± SD for the wound healing assay, statistical analysis by unpaired t-test; **p ≤ 0.01, ****p ≤ 0.0001.

Next, we studied whether the increased migration and resistance to cisplatin were associated with alterations in the signaling of PI3K-AKT-mTOR or RAS-MEK1/2-ERK1/2 pathways. In line with the unaltered inhibitor response, the PIK3R1 fusion did not affect the activity of the PI3K-AKT-mTOR pathway (Fig. 2A & Supplementary Fig. S7 A), in contrast to our expectation based on the protein function. However, the phosphorylation of native p85α was significantly decreased in the fusion-expressing cells (Fig. 2A and Supplementary Fig. S7 E) and less induced by epidermal growth factor (EGF) (Supplementary Fig. S7 B-D). Thus, although the fusion impairs the phosphorylation of native p85a, the effect is not associated with the downstream AKT or mTOR activity, as revealed by densitometric analysis of four biological replicates. In contrast, we found that ERK1/2 phosphorylation is significantly elevated in the cells with PIK3R1 fusion (Fig. 2B), which also showed resistance to a MEK1/2 inhibitor, trametinib (Fig. 1D). Similarly, an inhibitor of the upstream protein Ras, tipifarnib, led only to 1.4-fold decrease in the ERK1/2 phosphorylation in the fusion expressing cells, while in the vector control cells, the decrease was 5.2-fold (Fig. 2C). The results suggest that PIK3R1 fusion activates ERK1/2 in Ras-independent mechanism. Subsequently, we examined the association between migration and ERK activation by employing a wound healing assay with and without a specific ERK inhibitor, KO-947 (Fig. 2D). The significant decrease in migration by KO-947 suggests that the observed migration in the fusion cells is dependent on ERK1/2 activation. The finding is in line with a previous study demonstrating that a p85α truncating mutation is associated with increased invasion via ERK1/2 activation [27].

### *PIK3R1 fusion cells form rod and ring-like structures with CIN85 colocalization*

**Fig. 3:**
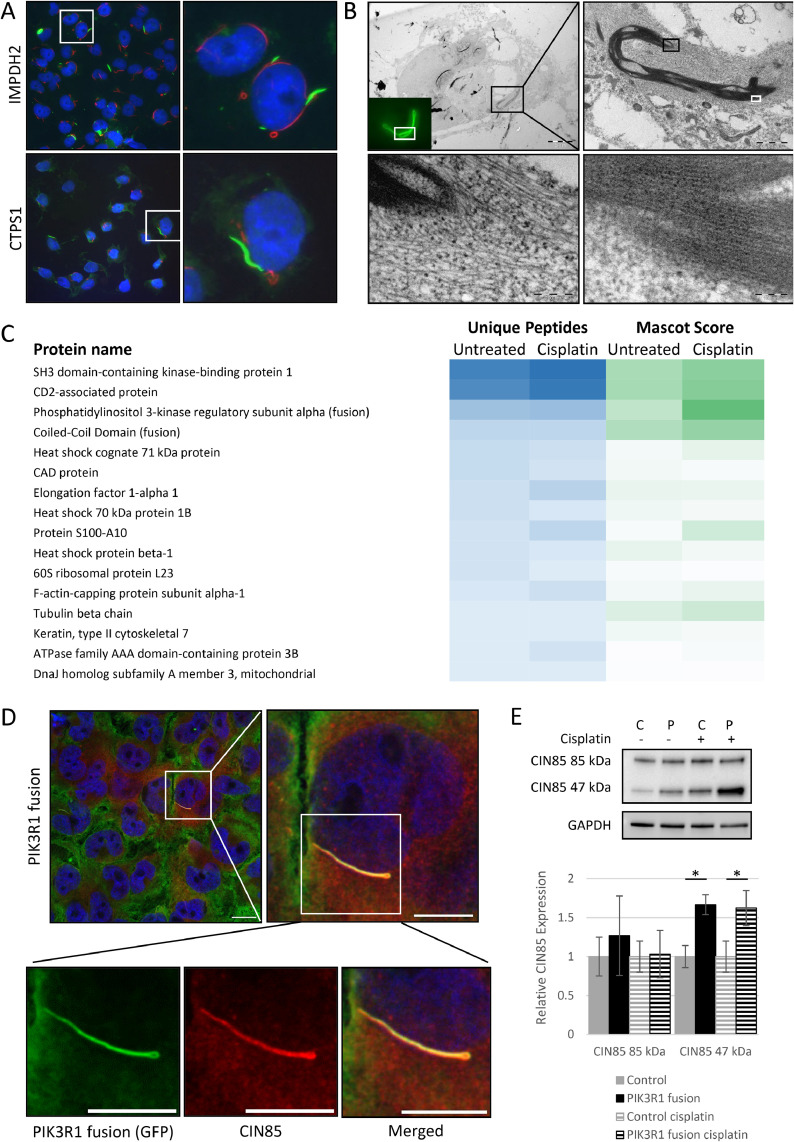
**Characterization of the PIK3R1 fusion protein complex.** A) Localization of PIK3R1 fusion protein complex. GFP-tagged PIK3R1-tagged fusion does not colocalize with rod and rings (RRs) visualized with IMPDH2 or CTPS1 (red), which were induced with 1 µmol/L MPA for 4 h and 100 µmol/L DON for 24 h. B) Correlative light-electron microscopy analysis of PIK3R1 fusion protein complex. PIK3R1 fusion cells were treated with 5 µmol/L cisplatin for 96 h to induce protein complexes. Fusion protein aggregates are visible perinuclearly close but do not colocalize with cellular organelles. Insets show the tail of the protein aggregate and ribosomes in the cytoplasm (magnification of the black box) and the filamentous structure of the PIK3R1 fusion protein complex (magnification of the white box). Scale bars in the electron microscopy images = 5 µm, 1 µm, 200 nm, and 100 nm, respectively. C) Sixteen most abundant proteins in the fusion structures using GFP-tag pull-down of untreated and cisplatin-induced structures. D) Fusion-expressing structures colocalize with CIN85. Cells were treated with 5 µmol/L cisplatin for 48 h, fixed, and stained with GFP (PIK3R1, green), CIN85 (red), and DNA (DAPI, blue). Scale bars of the first top two images represent 20 µm and for the three images below 10 µm. E) CIN85 47 kDa isoform is expressed in the fusion expression cells under normal culture conditions and after 3d cisplatin exposure. n = 3 Data are represented as mean ± SEM, statistical analysis by unpaired *t*-test; *p ≤ 0.05.

PIK3R1 fusion cells express dynamic protein complexes, which include the fusion protein and are increasingly expressed following cisplatin and trametinib treatments (Fig. 1D). These filamentous assemblies resemble rod and ring structures (RRs), *i.e*., cytoplasmic rod (10 µm in length), and ring (2-5 µm in diameter) -shaped assemblies, whose function is not fully understood [39]. The major components of RR are inosine monophosphate dehydrogenase 2 (IMPDH2) and CTP synthase 1 (CTPS1), whose inhibition by mycophenolic acid (MPA) or 6-Diazo-5-oxo-L-norleucine (DON) results in filament formation [44], [45], [46]. We induced their expression with MPA or DON to study whether the PIK3R1 fusion-expressing structures are RRs. The aggregated PIK3R1 fusion proteins are often localized in the vicinity of nuclei similar to RRs (Fig. 3A) but do not colocalize with them. The most noticeable difference is that the fusion protein aggregations are thicker in diameter. Correlative light-electron microscopy of the rod and ring-like structures showed that they closely associate with filaments that may be actin or intermediate filaments (Fig. 3B). Ribosome-like structures were visible between the filaments associated with the structures.

Next, we pulled down the GFP-tagged fusion protein with immunoprecipitation to uncover the protein composition of the RR-like structures and their putative role in the platinum resistance. Three biological replicates of vector control and PIK3R1 fusion were subjected to mass spectrometry; two without treatment and one with platinum treatment. As a result, 234 unique proteins (Supplementary Fig. S1 B) were identified between fusion expressing and control cell precipitates. Sixteen proteins were detected in all three fusion replicates and considered the most verifiable (Fig. 3C & Supplementary Fig. S1 C). In addition to PIK3R1 and the fusion-specific c-terminal peptide called “Coiled-Coil Domain,” the main functional protein groups included stress and protein folding related proteins (Heat shock protein beta-1; Elongation factor 1-alpha1; Heat shock cognate 71 kDa protein; DnaJ homolog subfamily A member 3, mitochondrial), and actin cytoskeleton-associated proteins (protein S100-A10, CD2-associated protein, Elongation factor 1-alpha1, F-actin-capping protein subunit alpha-1). CIN85 and CD2AP, along with the fusion peptides, had the highest unique peptide number and Mascot score.

Confocal microscopy confirmed the colocalization of the PIK3R1 fusion and CIN85 both after cisplatin exposure (Fig. 3D & Supplementary Fig. S8 A) and under standard culture conditions (Supplementary Fig. S8 B). In control and PIK3R1 fusion cells, confocal microscopy suggested that CIN85 expression increases after cisplatin exposure (Supplementary Fig. S8 A-B). Specifically, the protein expression of the CIN85 47 kDa isoform, but not the 85 kDa isoform, was significantly increased in the vehicle and cisplatin treated PIK3R1 fusion cells (Fig. 3E). CIN85 is known to negatively regulate p85α at its N-terminal SH3-domain [32], which is present in the fusion protein. Therefore, we further evaluated the association between CIN85 expression and ERK1/2 activation in the fusion-expressing cells. Analysis showed a parallel increase in CIN85 47 kDa isoform expression and ERK1/2 phosphorylation (Supplementary Fig. S9 & S3 C).

### *Rod and ring-like structure formation is associated with platinum resistance*

**Fig. 4:**
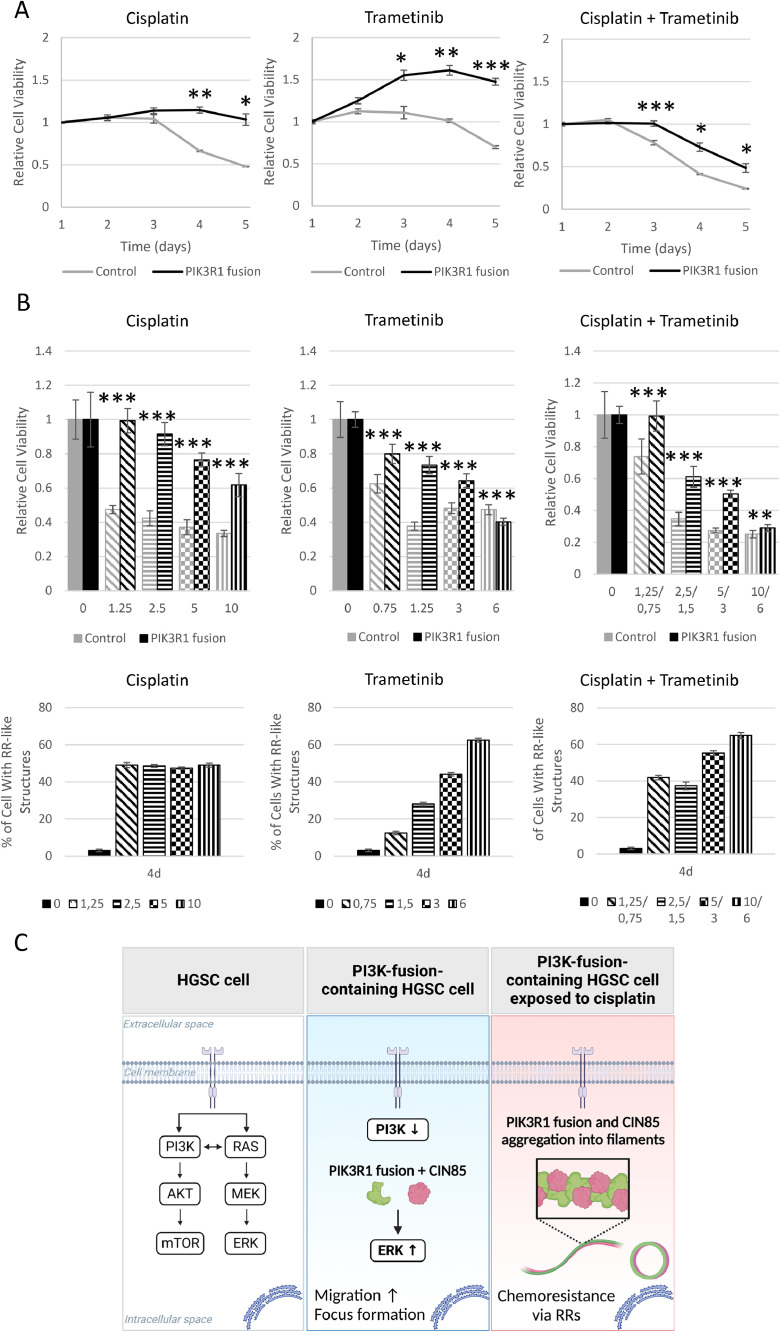
**Rod and ring-like structure formation is associated with cell survival and treatment resistance.** A) PI3KR1 fusion-expressing cells maintained viability under cisplatin and trametinib treatment (MTS assay). The viability of the treated cells is normalized to the vehicle and 1-day cell viability. B) Upper panel: Relative cell viability of control and fusion cells at different concentrations of cisplatin and trametinib and their combination on the fourth treatment day. Lower panel: Cisplatin and trametinib induced RR-like structures in fusion-expressing cells at different concentrations on day four. The data are represented from three biological replicates (mean ± SD). Unpaired t-test; *p ≤ 0.05, **p ≤ 0.01, ***p ≤ 0.001, ****p ≤ 0.0001. C) Graphical illustration of events leading to chemoresistance. PIK3R1 fusion (middle panel) enhances malignant phenotype (e.g., migration) via ERK1/2 activation either directly or indirectly. In addition, wild-type PIK3R1/p85α expression is decreased. CIN85 potentially inhibits fusion protein activity by binding on the SH3 domain of the PIK3R1 fusion. Under cisplatin treatment (right panel), PIK3R1 fusion aggregates with CIN85 resulting in filamentous RR-like structure formation and simultaneous chemoresistance. Created with BioRender.com.

To evaluate whether the RR-like structures are associated with platinum resistance, we performed a parallel investigation of cell viability and the RR-like structure formation under 5 µmol/L cisplatin and 3 µmol/L trametinib. We first verified that the PIK3R1 fusion cells maintained their proliferation during five-day cisplatin exposure, whereas 52 % of the vector control cells had died on day five. In addition, PIK3R1 fusion cells showed significant resistance to trametinib. However, PIK3R1 fusion cells were sensitive to the combination treatment (Fig. 4A & Supplementary Fig. S3 B & S10 A). These findings were associated with strong induction of RR-like structure formation (Supplementary Fig S10 B). We subsequently examined whether varying concentrations had an effect on cell viability and the formation of structures. Control and fusion cells were exposed to four different concentrations of cisplatin and trametinib, as well as their combination. The cell viability and the number of structures were then counted for four days. The cell viability at various concentrations was consistent with that observed at a single concentration: fusion cells exhibit resistance to cisplatin across all concentrations but remained sensitive to combination treatment (Fig. 4B upper panel). Sub-lethal concentrations induce RR-like structure formation, with a more pronounced effect observed with cisplatin compared to trametinib. Approximately 49 % of the fusion cells expressed structures following cisplatin treatment, regardless of the concentration, while the number of structures increased with the ascending concentrations of trametinib during treatment (Fig. 4B lower panel).

## Discussion

In HGSC, platinum resistance can occur with multiple mechanisms, most likely varying from cell to cell within each heterogeneous tumor. The cells with acquired resistance mechanism are likely to be enriched during tumor evolution leading to chemotherapy failure. Extensive structural genomic alterations, including gene fusions, are typical for HGSC. However, the mechanisms underlying the fusion genes’ involvement in chemoresistance are poorly characterized. Here, we characterize a novel mechanism, in which a PIK3R1 fusion paradoxically activates the ERK1/2 pathway, increases ovarian cancer cell migration and chemoresistance, and thereby potentially facilitates metastatic dissemination.

We expected that the PIK3R1 fusion would affect PI3K-AKT-mTOR signaling. p85α/PIK3R1 is a negative pathway regulator, and its disruption is likely to lead to increased pathway activity and downstream tumor-promoting mechanisms. However, the PI3K-AKT-mTOR pathway was intact in the fusion-expressing cells. Instead, the mechanism behind the PIK3R1 fusion-driven chemoresistance and increased cell migration is associated with elevated ERK1/2 activation, CIN85 47 kDa isoform expression, and formation of RR-like structures (Fig. 4C). ERK1/2 is known to participate in multiple oncogenic mechanisms and treatment resistance [2,47]. Interestingly, ERK1/2-mediated cell invasion is also increased by *PIK3R1* gene mutations, which cause a truncated p85α similar to the PIK3R1 fusion [27].

CIN85 is a pro-oncogenic scaffold protein that can mediate various molecular mechanisms through its physical interaction with other proteins [48]. It is involved in multiple cancer cell-promoting functions [33,48], and its upregulation is associated with the advanced cancer stage and lymph node metastasis [33][37][38]. CIN85 also activates RAS-MEK1/2-ERK1/2 signaling [33], leading to enhanced cell adhesion, migration, and invasive behavior [34], [35], [36]. In our study, we noticed that CIN85 expression is particularly increased in fusion expressing cells after cisplatin treatment suggesting that CIN85 may also be involved in mechanisms of chemoresistance.

Interestingly, platinum-induced RR-like structure formation in the PIK3R1 fusion cells. The mass spectrometry analysis of the RR-like structures expressing the fusion protein revealed that the main components in the structures are the fusion protein, CIN85, and four proteins associated with stress and protein folding. Thus, the structure formation may result from platinum-induced cellular stress and the expression of the misfolded fusion protein. In addition, the binding and negative regulation of CIN85 on fusion protein´s SH3 domain may lead to protein aggregation and RR-like structure formation. The number and size of the structures were associated with the CIN85 expression levels, ERK1/2 activation, and treatment resistance. In addition, ERK1/2 activation was linked with oncogenic phenotype, and its upstream inhibition with trametinib resulted in treatment resistance and structure formation.

Analogously, in the RR formation, the IMPDH2 aggregation is previously shown to be a resistance mechanism to the antiviral nucleotide synthesis inhibitor ribavirin. IMPDH2 and CTPS2 are the two critical regulators of *de novo* purine and pyrimidine nucleotide synthesis and the major components of RRs [44], [45], [46]. Aggregation of IMPDH2 into RRs adjusts its allosteric regulation, makes IMPDH2 less sensitive to feedback inhibition, and ensures the guanine nucleotide synthesis for the rapidly proliferating cell [49], [50], [51]. We hypothesize that our fusion-driven structure formation is a similar cell survival mechanism. However, further studies are needed to enlighten the specific mechanism of the RR-like structure-mediated platinum and trametinib resistance and whether the mechanism is more general in drug resistance.

The urgency to identify treatment options for recurrent cancers based on genomic alterations is increasing. Our study emphasizes that an intuitive treatment option based on the key functions of the fusion partners may not be effective, as demonstrated here with poor response to PI3K-pathway inhibitors. Our results underscore the importance of functionally characterizing the consequences of identified genomic alterations and considering alternative oncogenic or regulatory activities for targeted treatments.

## Funding

This work was supported by the Turku University Foundation; The Finnish Cultural Foundation; The Drug Research Doctoral Program at the University of Turku; The Instrumentarium Science Foundation; and The European Union's Horizon 2020 research and innovation program DECIDER [965193].

## CRediT authorship contribution statement

**Heidi Rausio:** Conceptualization, Formal analysis, Funding acquisition, Investigation, Methodology, Project administration, Validation, Writing – original draft. **Alejandra Cervera:** Conceptualization, Methodology, Software. **Vanina D. Heuser:** Investigation, Supervision. **Gun West:** Supervision. **Jaana Oikkonen:** Resources, Writing – review & editing. **Elena Pianfetti:** Software. **Marta Lovino:** Software. **Elisa Ficarra:** Software. **Pekka Taimen:** Resources. **Johanna Hynninen:** Resources, Writing – review & editing. **Rainer Lehtonen:** Conceptualization. **Sampsa Hautaniemi:** Conceptualization, Funding acquisition, Writing – review & editing. **Olli Carpén:** Conceptualization, Funding acquisition, Project administration, Supervision, Writing – review & editing. **Kaisa Huhtinen:** Conceptualization, Funding acquisition, Project administration, Supervision, Writing – review & editing.

## Declaration of competing interest

The authors declare that they have no known competing financial interests or personal relationships that could have appeared to influence the work reported in this paper.

## Appendix Supplementary materials

Image, application 1

Image, application 2

Image, application 3
