# Supplementary material for: PIK3R1 fusion drives chemoresistance in ovarian cancer by activating ERK1/2 and inducing rod and ring-like structures
Source: Neoplasia. 2024 Mar 14;51:100987. doi: 10.1016/j.neo.2024.100987 (PMC10955102; doi:10.1016/j.neo.2024.100987)
Supplement: Supplementary file 1 [file mmc1.pdf]

Supplementary Figure S1

OVCAR-8 transfected cells

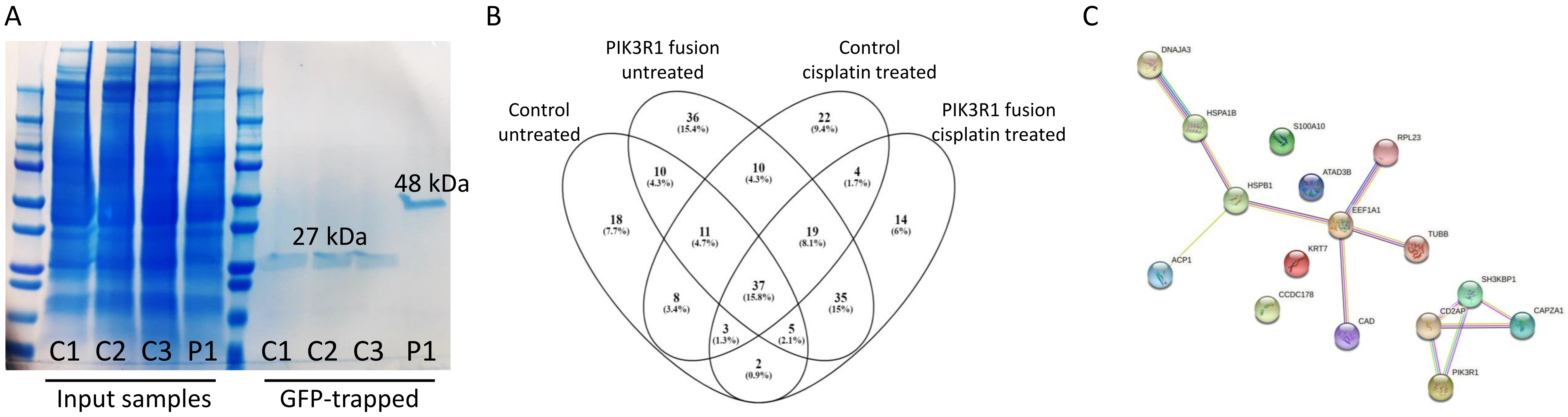

Figure S1. Protein analysis. A) PageBlue-staining of the immunoprecipitated control and PIK3R1 fusion samples. The total protein extracted from the control and PIK3R1 fusion cells is shown as input samples, and the immunoprecipitated proteins are shown as GFP-trapped. C1-C3 = Control cells with three replicates, and P1 = One replicate of the PIK3R1 fusion cells. B) Venn diagram of untreated and cisplatin-treated control and PIK3R1 fusion cells. Three technical replicates from two independent experiments were analyzed. C) STRING Database Version 11.5 was used to predict the protein-protein interactions of the 16 proteins discovered from the PIK3R1 fusion cells detected by mass spectrometry analysis.

Supplementary Figure S2

Patient ovarian tissue

A B

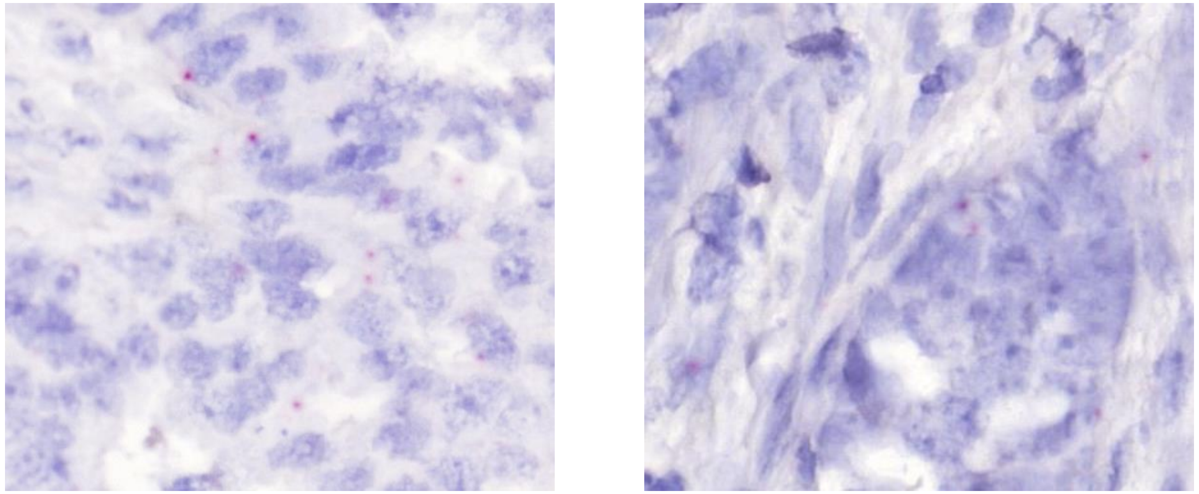

Figure S2. PIK3R1 fusion expression in the patient tumor specimens. RNA *in situ* of the fusion-specific probe localizes the signal (red dots) in the tumor cells of the right ovary (A) and in metastasis of a right para-aortic lymph node (B).

Supplementary Figure S3

HEK293 transfected cells

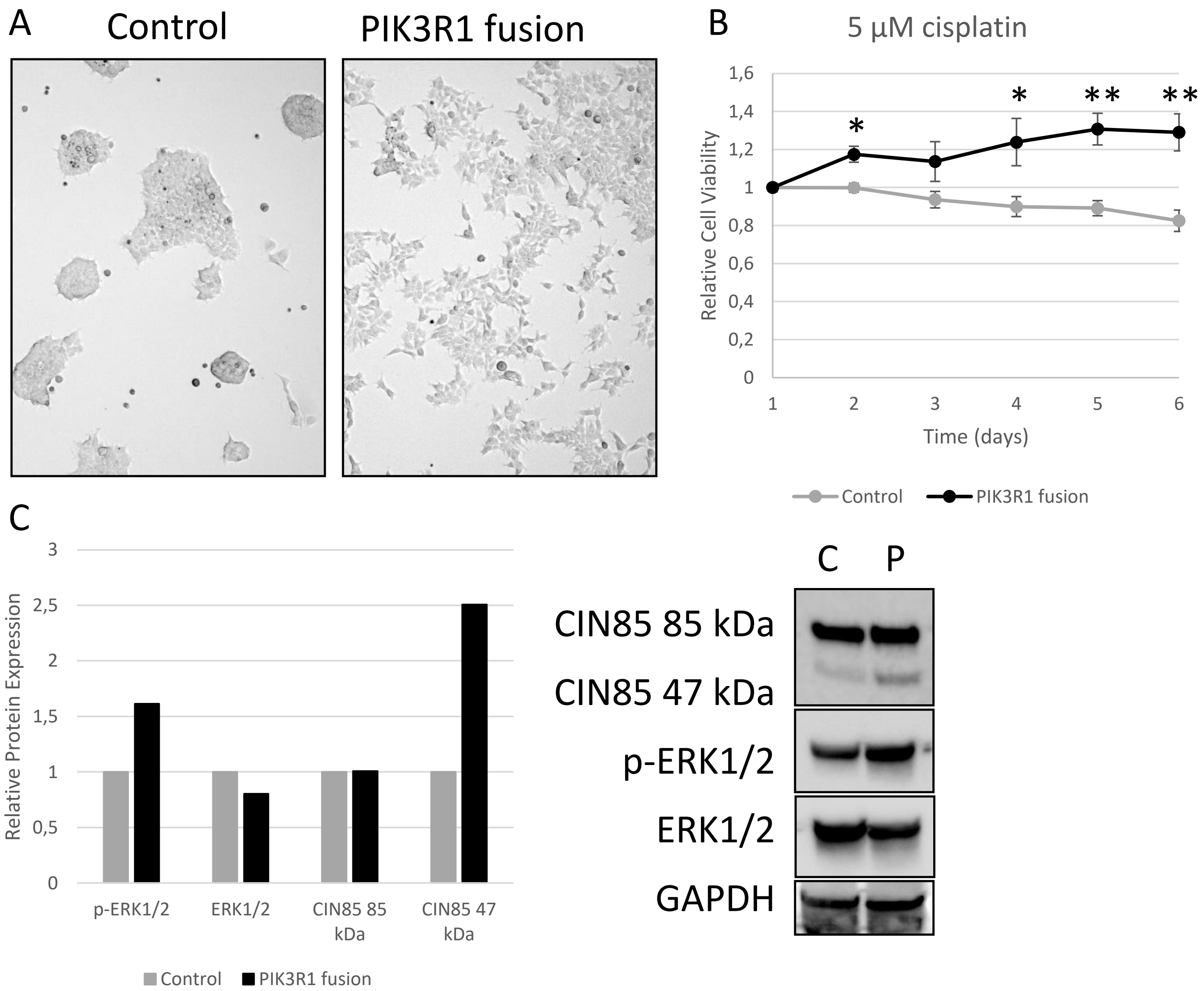

Figure S3. A) Morphologies of control and PIK3R1 fusion (HEK293) cells under 10x magnification by phase contrast microscopy. PIK3R1 fusion cells form unorganized populations with unclear cell borders, while vector-expressing control cells grow in round, compact colonies. B) 10 000 cells were plated and incubated overnight. Control and PIK3R1 fusion cells were treated with 5 µM cisplatin for 5 d. Cell viability was determined every 24 h for five days by MTS assay. Treated cells are normalized to the vehicle and 1 d time point. Statistical analysis by unpaired *t*-test; *$p \leq 0.05$, **$p \leq 0.01$. Error bars represent ±SEM. C) Western blot analysis of CIN85 and ERK1/2 expression from control and PIK3R1 fusion cells under standard culture conditions.

Supplementary Figure S4

OVCAR-8 transfected cells

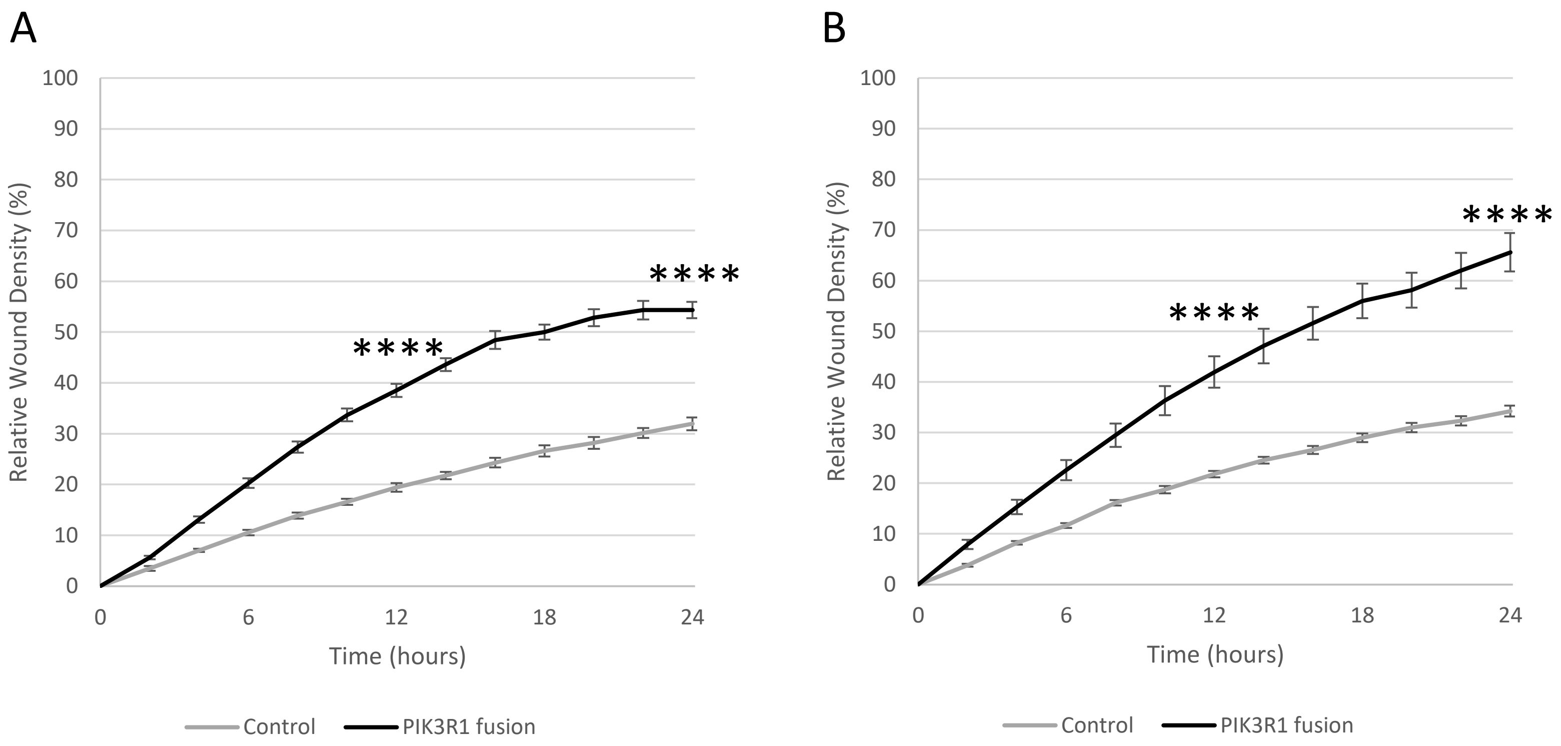

Figure S4. Wound healing assays. A) Second repetition of migration from the control and PIK3R1 fusion cells detected by wound healing assay. n = 8 for control and n = 12 for PIK3R1 fusion cells. B) Third repetition of migration from the control and PIK3R1 FUSION cells detected by wound healing assay. n = 24 for control and n = 15 for PIK3R1 fusion cells. Data are normalized to 0 h timepoint. Statistical analysis by unpaired t-test; ****$p \leq 0.0001$. Error bars represent ±SEM.

Supplementary Figure S5

OVCAR-8 transfected cells

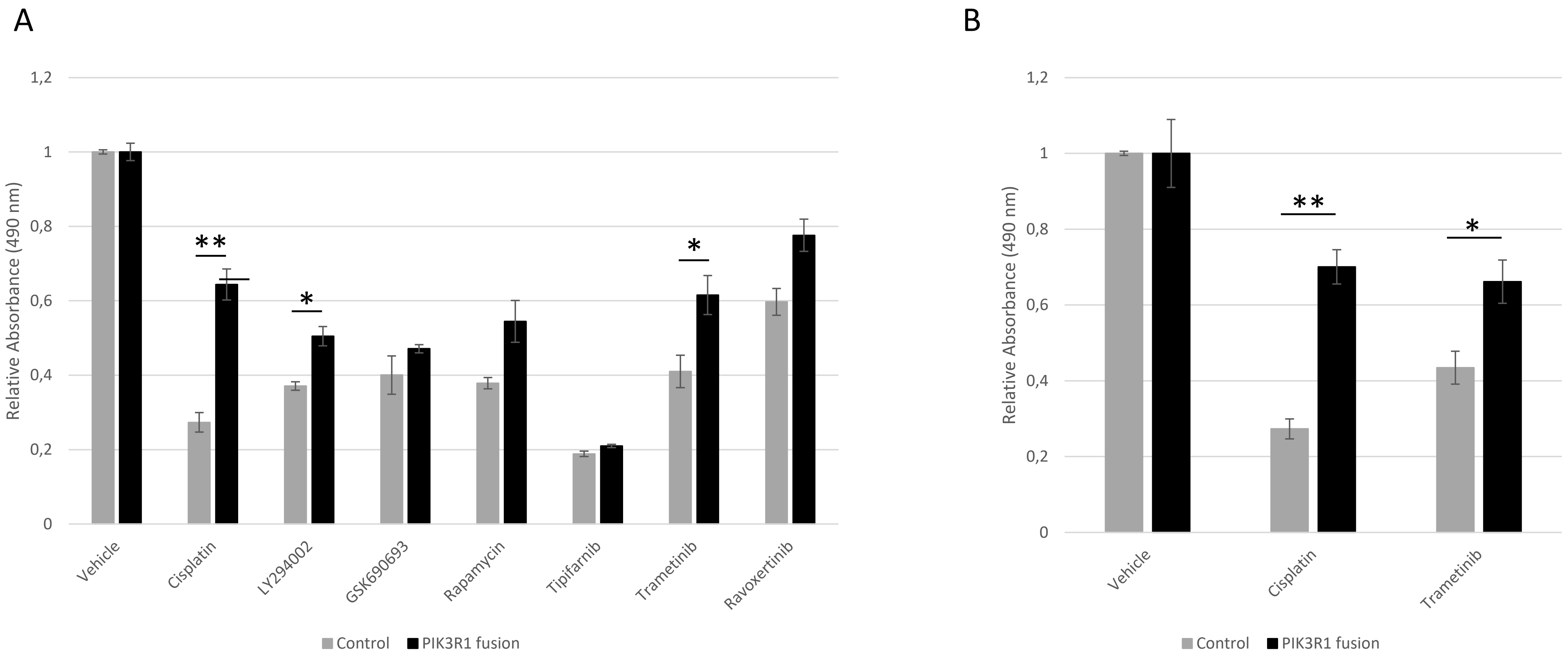

Figure S5. Colony formation assays. A) Second repetition of the colony formation assay of control and PIK3R1 fusion cell lines. Cells were grown 8 d without treatment or treated with 5 µM cisplatin, 2 µM LY294002, 12 µM GSK690693, 1 µM rapamycin, 7 µM tipifarnib, 5 µM trametinib and 3 µM ravoxertinib for 3 d starting on day 5. B) Third repetition of the colony formation assay of control and PIK3R1 fusion cell lines. Cells were grown for 8 d without treatment or treated with 5 µM cisplatin and 5 µM trametinib for 3 d starting day 5. Data are normalized to the vehicle. The mean of the three technical replicates is shown. Statistical analysis by unpaired *t*-test; *$p \leq 0.05$, **$p \leq 0.01$. Error bars represent ±SEM.

Supplementary Figure S6

OVCAR-8 transfected cells

Figure S6. Fluorescence microscopy analysis of RR-like structure formation in the PIK3R1 fusion cells under 10x magnification. Control and PIK3R1 fusion cells were grown 8 d without treatment or treated with cisplatin, LY294002 (PI3Ki), GSK690693 (pan-AKTi), rapamycin (mTORi), tipifarnib (Rasi), trametinib (MEKi) and ravoxertinib (ERKi) for 3 d starting on day 5.

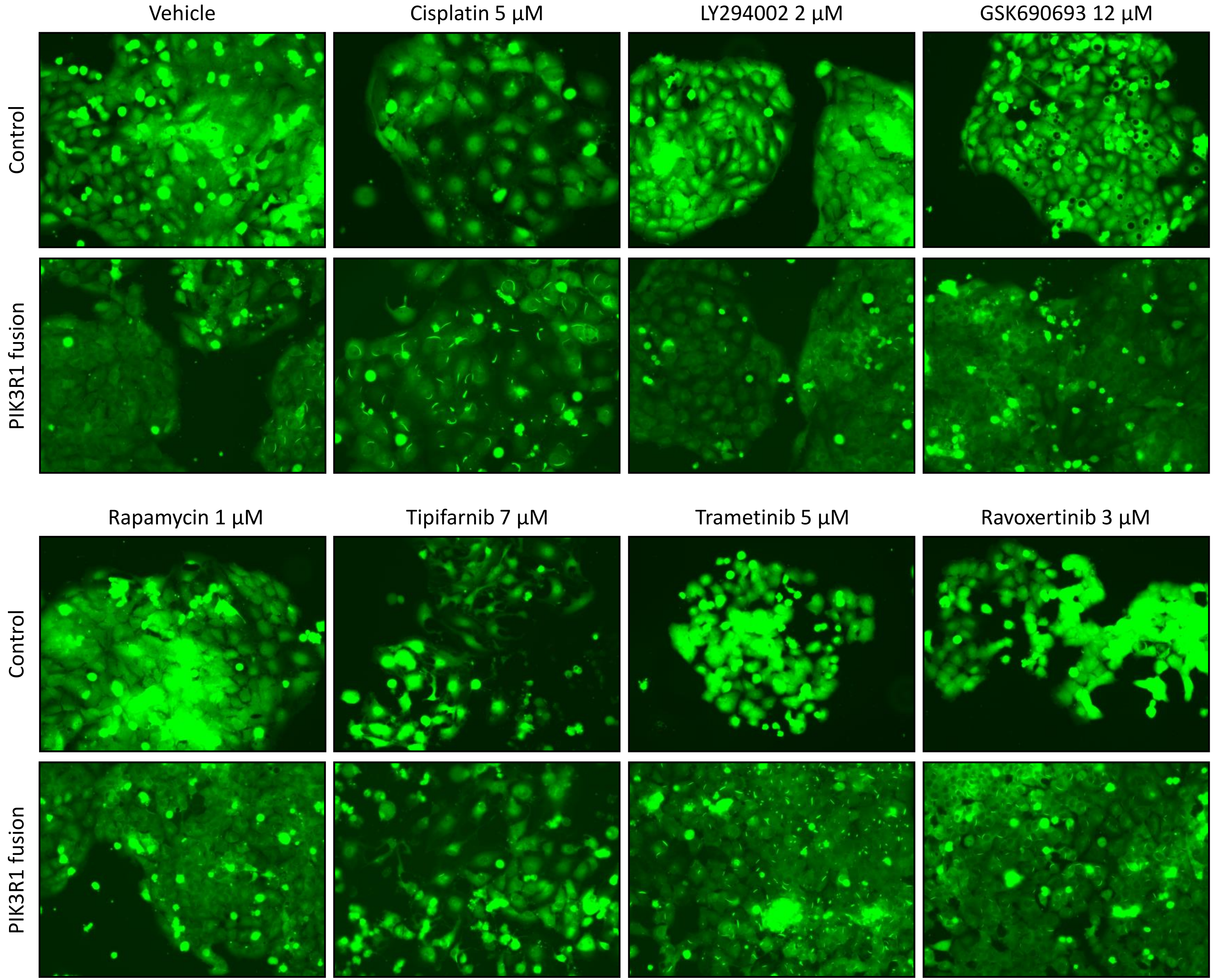

Supplementary Figure S7

OVCAR-8 transfected cells

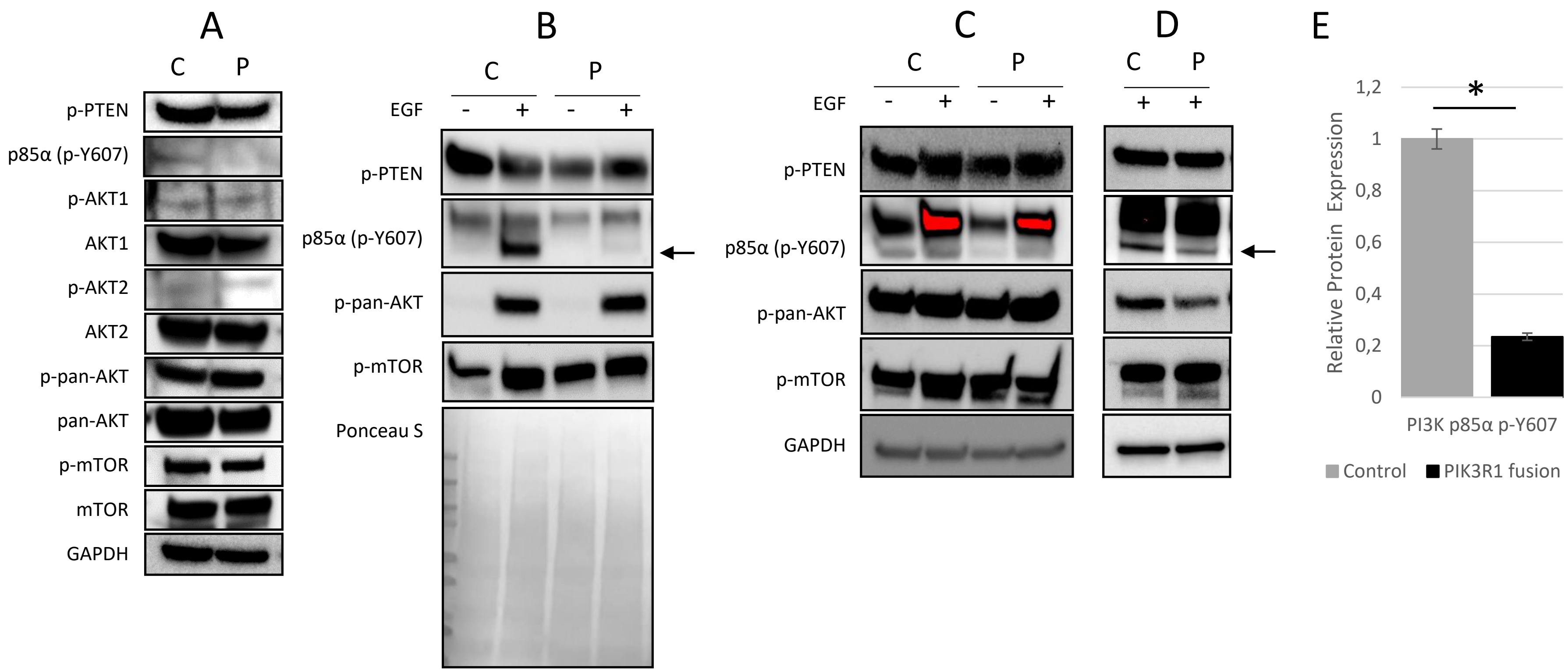

Figure S7. Protein expression in PI3K-AKT-mTOR pathway. A) Second repetition of the Western blot analysis PI3K-AKT-mTOR pathway protein expression from the control and PIK3R1 fusion cells under standard culture conditions. B-D) Three independent repetitions of the Western blot analysis of from control and PIK3R1 fusion cells induced with 50 ng/ml EGF for 1 h. The two black arrows indicate the correct size of the native p85α (p-Y607). PIK3R1 fusion protein lacks the Y607 phosphorylation site. C = Control and P = PIK3R1 fusion cells. E) The relative expression of p85α (p-Y607) from three biological replications. Statistical analysis by unpaired *t*-test; $*p \leq 0.05$. Error bars represent ±SEM.

Supplementary Figure S8 A

OVCAR-8 transfected cells

Figure S8 A. Colocalization of CIN85 with PIK3R1 fusion protein by confocal microscopy. B) Cells were treated with 5 µM cisplatin for 48 h, fixed and stained with GFP (PIK3R1 fusion, green), CIN85 (red) and DNA (DAPI, blue). Representative middle Z-stack sections are shown. Scale bars of the first images on the left-hand column represent 20 µm, and for the other images, 10 µm.

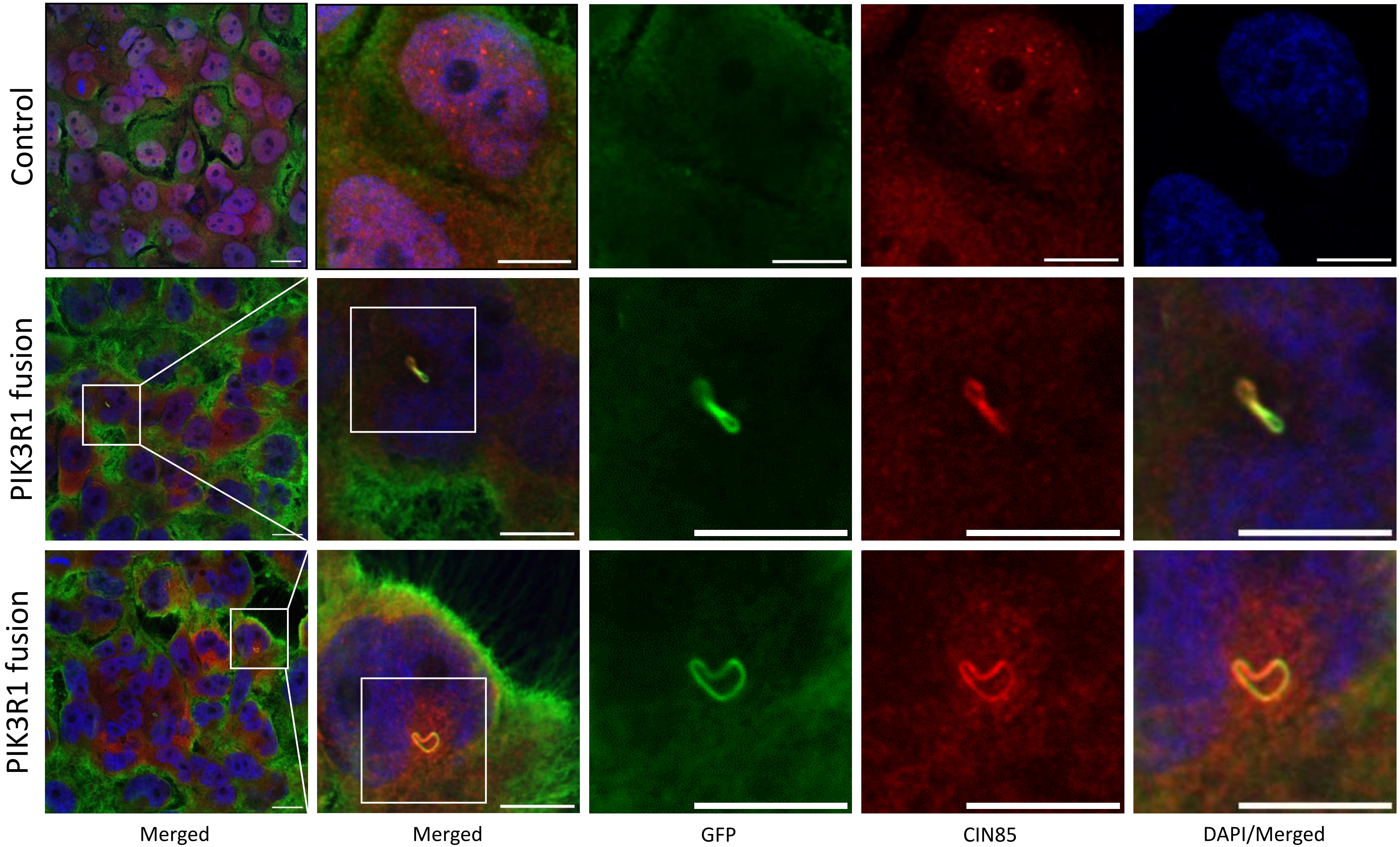

Supplementary Figure S8 B

OVCAR-8 transfected cells

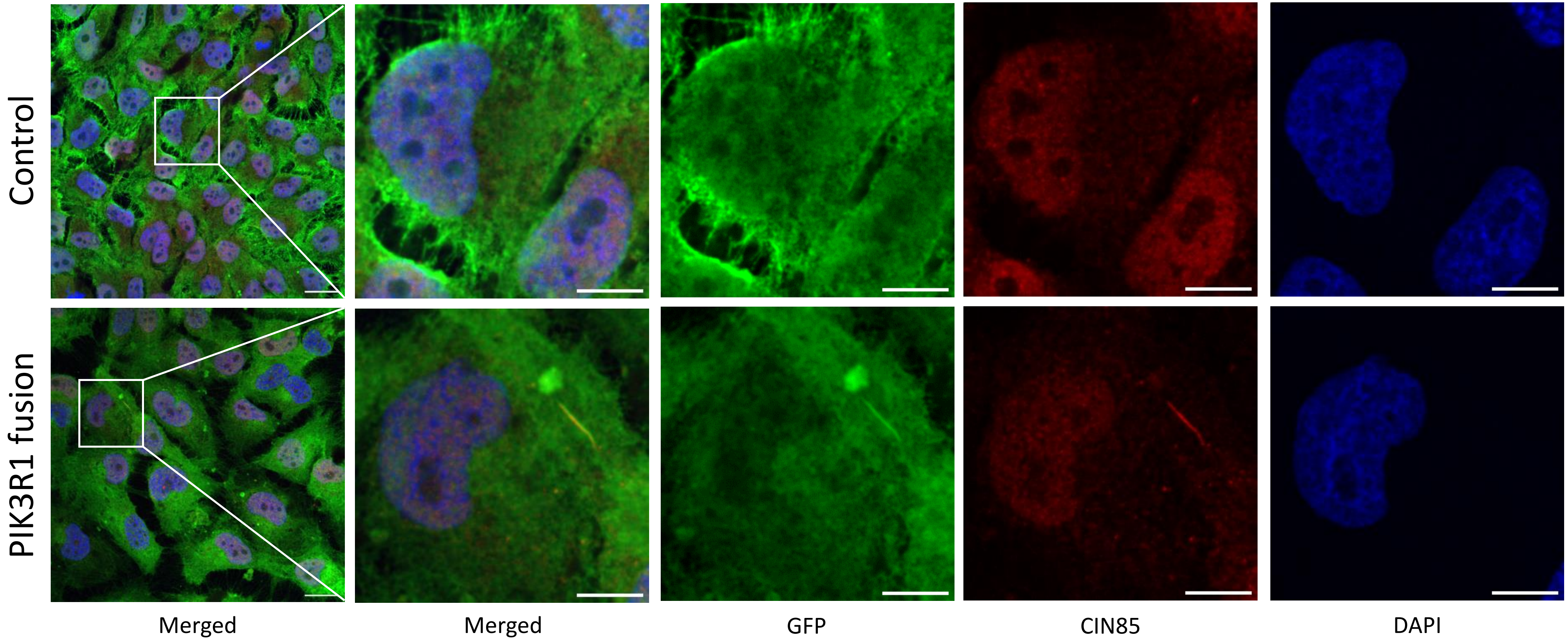

Figure S8 B. Colocalization of CIN85 with PIK3R1 fusion protein by confocal microscopy. A) Control and PIK3R1 fusion cells under standard culture conditions. Cells were fixed and stained with GFP (PIK3R1 fusion, green), CIN85 (red) and DNA (DAPI, blue). Representative middle Z-stack sections are shown. Scale bars of the first images on the left-hand column represent 20 μm, and for the other images, 10 μm.

Supplementary Figure S9

OVCAR-8 transfected cells

Figure S9. Western blot analysis of CIN85 and ERK1/2 expression from control and PIK3R1 fusion cells under normal culture conditions. *n* = 3 Data are represented as mean ± SEM, statistical analysis by unpaired *t*-test; **$p \leq 0.01$.

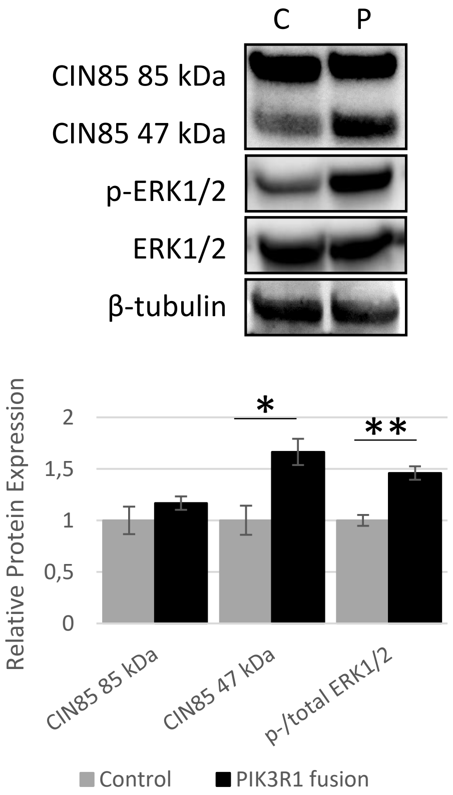

Supplementary Figure S10

OVCAR-8 transfected cells

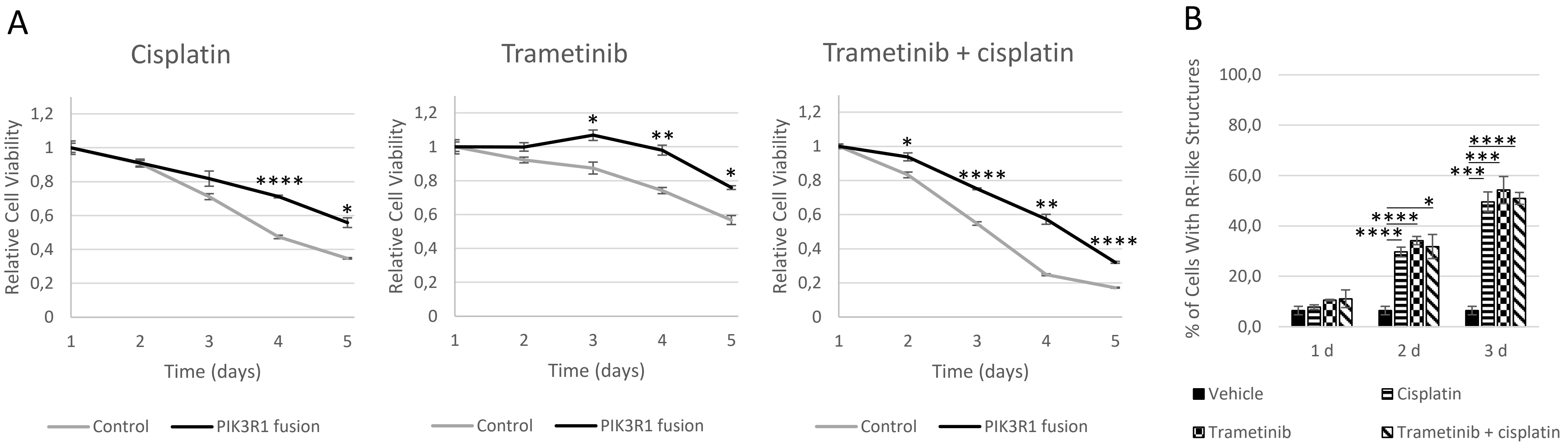

Figure S10. A) In the second repetition of the MTS assay, 5000 cells were plated and incubated overnight. Control and PIK3R1 fusion cells were treated with 5 µM cisplatin, 3 µM trametinib, and a combination of 5 µM cisplatin and 3µM trametinib for 5 d. Cell viability was determined every 24 h for five days by MTS assay. Treated cells are normalized to 1 d time point. B) Cisplatin and trametinib induce the RR-like structures in the fusion-expressing cells linearly for three days (average ±SEM). A range of 63 to 269 cells was counted at each time point. Only 6% of the untreated PIK3R1 fusion cells expressed RR-like structures, while the number of the structure-expressing cells increased in a time-dependent manner. After 48 h and 72 h cisplatin exposure, 32% and 52% of the PIK3R1 fusion cells expressed RR-like structures, and after trametinib exposure, 34% and 54% of cells expressed RR-like structures, respectively. Corresponding percentages after combination treatment were 32% and 51% Statistical analysis by unpaired *t*-test; *$p \le 0.05$, **$p \le 0.01$, ***$p \le 0.001$, ****$p \le 0.0001$. Error bars represent ±SEM.
