# Supplementary material for: PIK3R1 fusion drives chemoresistance in ovarian cancer by activating ERK1/2 and inducing rod and ring-like structures
Source: Neoplasia. 2024 Mar 14;51:100987. doi: 10.1016/j.neo.2024.100987 (PMC10955102; doi:10.1016/j.neo.2024.100987)
Supplement: Supplementary file 2 [file mmc2.pdf]

Supplementary Methods

Table S1. List of the inhibitors

Inhibitor	Concentrations colony assay	Concentrations MTS assay	Concentrations WB	Concentrations IF
Cisplatin	5 µM	5 µM	5-10 µM	5 µM
LY294002 (pan-PI3K)	2 µM			
GSK690693 (pan-AKT)	12 µM			
Rapamycin (mTOR)	1 µM			
Tipifarnib (RAS)	7 µM		10 µM	
Trametinib (MEK1/2)	5 µM	3 µM	3-10 µM	
Ravoxertinib (ERK1/2)	3 µM			
Mycophenolic acid (MPA)				1 µM
6-diazo-5-oxo-norleucine (DON)				100 µM

Table S2. List of primary and secondary antibodies

Antibodies	Application	Company	Catalog number	Dilution
p-PTEN	WB	Cell Signaling	9549	1:1000
Phospho-PI3 Kinase p85	WB	Abcam	ab182651	1:1000
PI3 Kinase p85	WB	Invitrogen	PA5-32550	1:1000
Phospho-AKT1	WB	Cell Signaling	9018	1:1000
AKT1	WB	Cell Signaling	2938	1:1000
Phospho-AKT2	WB	Cell Signaling	8599	1:1000
AKT2	WB	Cell Signaling	3063	1:1000
Phospho-pan-AKT	WB	Cell Signaling	4060	1:1000
pan-AKT	WB	Cell Signaling	4691	1:1000
Phospho-mTOR	WB	Cell Signaling	5536T	1:1000
mTOR	WB	Cell Signaling	2983T	1:1000
IMPDH2	IF	Atlas antibodies	HPA001400	1:500
CTPS1	IF	Atlas antibodies	HPA051322	1:200
Phospho-c-Raf	WB	Cell Signaling	9427	1:1000
Phospho-MEK1/2	WB	Cell Signaling	9154	1:1000
Phospho-p44/42 MAPK (ERK1/2)	WB	Cell Signaling	4370	1:1000
p44/42 MAPK (ERK1/2)	WB	Cell Signaling	4695	1:1000
CIN85	IF, WB	Santa Cruz Biotechnology	sc-166862	IF; 1:50, WB; 1:1000
GAPDH	WB	ThermoFisher	MA5-31457	1:5000
GAPDH 488	WB	Proteintech	CL488-60004	1:5000
β-tubulin	WB	Proteintech	CL488-66240	1:5000
GFP	IF	Santa Cruz Biotechnology	sc-9996	1:200 - 1:500
GFP	IF	Novus Biologicals	NB600-308	1:200 - 1:500
anti-mouse Alexa Fluor 488	IF	Life Technologies	A11059	1:400
anti-rabbit Alexa Fluor 488	IF	Invitrogen	A11001	1:400

anti-mouse Alexa Fluor 555	IF	Life Technologies	A21427	1:400
anti-mouse Alexa Fluor 647	IF	Invitrogen	A21236	1:400
HRP-conjugated rabbit anti-mouse	WB	Dako Denmark A/S	P0161	1:5000
HRP-conjugated swine anti-rabbit	WB	Dako Denmark A/S	P0217	1:5000

RNA *in situ* hybridization

RNA *in situ* hybridization with BaseScope assay was carried out to visualize PIK3R1 fusion gene expression according to the manufacturer's instructions (Advanced Cell Diagnostics, Newark, CA, USA). We utilized customized BaseScope probes for PIK3R1-CCDC178 (#719681) fusion, as well as BaseScope Positive Control Probe Hs-PPIB-1ZZ (#701041) and Negative Control Probe-DapB-1ZZ (#701021). In brief, formalin-fixed paraffin-embedded tissue sections were deparaffinated, treated with target retrieval reagents at 98 °C for 15 min, and digested with protease IV for 15 min at 40 °C in the hybridization oven. The slides were next hybridized with the BaseScope probes for 2 h at 40 °C, followed by serial amplification steps at 40 °C in the hybridization oven or at room temperature as instructed, and finally incubated with the Fast-Red substrate at room temperature to visualize the hybridization signals. The stained slides were digitalized using a 3DHISTECH Pannoramic 250 FLASH II digital slide scanner at the Genome Biology Unit supported by HiLIFE and the Faculty of Medicine, The University of Helsinki and Biocenter Finland.
